# Supplementary material for: Gender inequalities of psychosomatic complaints at work vary by occupational groups of white- and blue-collar and level of skill: A cross sectional study
Source: PLoS One. 2024 Jul 11;19(7):e0303811. doi: 10.1371/journal.pone.0303811 (PMC11239076; doi:10.1371/journal.pone.0303811)
Supplement: S1 Data — (DOCX) [file pone.0303811.s001.docx]

# Supporting Information

## Data access.

This paper uses data from the BIBB/BAuA Employment Survey of the Working Population on Qualification and Working Conditions in Germany 2018. The survey was conducted by the Federal Institute for Vocational Education and Training (BIBB), and the Federal Institute for Occupational Safety and Health (BAuA). The data is owned by this third-party. Thus, the data is not uploaded to PLOS as the authors are not the owners of the data but only users. Thus, the authors can not legally distribute the data to PLOS. The data were included in the data package with data in STATA- and SPSS format, the questionnaires and field reports called “ZA4820 - BIBB/BAuA-Employment Survey 2006”. The data access was provided via a Scientific-Use-File (SUF) of the Data Research Centre at the Federal Institute for Vocational Training and Education (BIBB-FDZ). The dataset is freely available for academic purposes in a public repository on request after having signed an agreement with the owner (further information available online via <https://www.bibb.de/de/1403.php>). The use of the data is subject to guidelines and requires a formal application to the BIBB-FDZ. SUFs are distributed directly via the BIBB-FDZ. For this purpose, an application form (available online via <https://www.bibb.de/dokumente/pdf/BIBB_FDZ_Antrag_SUF_FDZ_deutsch.pdf>) must be completed, signed and sent to the BIBB-FDZ by post (address: BIBB - Bundesinstitut für Berufsbildung Arbeitsbereich 1.5: Forschungsdatenzentrum, Postfach 20 12 64, 53142 Bonn, Germany) or e-mail (address: [fdz@bibb.de](mailto:fdz@bibb.de)). Data access and permission to use for research has been granted by the BIBB-FDZ to the researchers Johannes Beller and Julia Graßhoff after signing the agreement.
