# Supplementary material for: Gender inequalities of psychosomatic complaints at work vary by occupational groups of white- and blue-collar and level of skill: A cross sectional study
Source: PLoS One. 2024 Jul 11;19(7):e0303811. doi: 10.1371/journal.pone.0303811 (PMC11239076; doi:10.1371/journal.pone.0303811)
Supplement: Attachment — Submitted filename: Rebuttal_letter_Response_to_Reviewers.docx [file pone.0303811.s002.docx]

# **Rebuttal letter / Response to Reviwers**

Dear Sir or Madam,

Thank you for your invitation to revise and resubmit our manuscript. We appreciate the constructive comments and suggestions provided. We tried to address and incorporate all points raised to improve the manuscript. In the following, we quote the points raised by the two reviewers and describe what we adjusted in the revised version of our manuscript.

Best regards,
Julia Graßhoff

**Journal requirements:**
**Comment 1 Journal*:*** *Please ensure that your manuscript meets PLOS ONE's style requirements, including those for file naming. The PLOS ONE style templates can be found at https://journals.plos.org/plosone/s/file?id=wjVg/PLOSOne_formatting_sample_main_body.pdf and* [*https://journals.plos.org/plosone/s/file?id=ba62/PLOSOne_formatting_sample_
title_authors_affiliations.pdf*](https://journals.plos.org/plosone/s/file?id=ba62/PLOSOne_formatting_sample_title_authors_affiliations.pdf)
**Answer 1 Journal:** We have adjusted the first page including the author section according to the PLOS ONE style requirements.

**Comment 2 Journal:** *Please state what role the funders took in the study. If the funders had no role, please state: ""The funders had no role in study design, data collection and analysis, decision to publish, or preparation of the manuscript."" If this statement is not correct you must amend it as needed. Please include this amended Role of Funder statement in your cover letter; we will change the online submission form on your behalf.*
**Answer 2 Journal:** We have included the latter answer option in the cover letter.

**Comment 3 Journal:** *All PLOS journals now require all data underlying the findings described in their manuscript to be freely available to other researchers, either 1. In a public repository, 2. Within the manuscript itself, or 3. Uploaded as supplementary information. This policy applies to all data except where public deposition would breach compliance with the protocol approved by your research ethics board. If your data cannot be made publicly available for ethical or legal reasons (e.g., public availability would compromise patient privacy), please explain your reasons on resubmission and your exemption request will be escalated for approval.*
**Answer 3 Journal:** We have adjusted the following additional information in the methods section to clarify (changes highlighted in green): “As the data are owned by this third party, the authors are not the data owners, but only users. The dataset is available for academic purposes. All underlying data are freely available in a public repository on request after having signed an agreement with the owner (<https://www.bibb.de/de/1403.php>). SUFs are distributed directly via the BIBB-FDZ. For this purpose, an application form (available online via <https://www.bibb.de/dokumente/pdf/BIBB_FDZ_Antrag_SUF_FDZ_deutsch.pdf>) must be completed, signed and sent to the BIBB-FDZ by post (address: BIBB - Bundesinstitut für Berufsbildung Arbeitsbereich 1.5: Forschungsdatenzentrum, Postfach 20 12 64, 53142 Bonn, Germany) or e-mail (address: [fdz@bibb.de](mailto:fdz@bibb.de)). Data access and permission to use for research has been granted by the BIBB-FDZ to the researchers Johannes Beller and Julia Graßhoff.”

**Comment 4 Journal**: *Please include a copy of Table 3 which you refer to in your text on page.*
**Answer 4 Journal**: The table is already included in the paper but there may have been a confusion as the style requirements of PLOS ONE require the table to be put right under the referring paragraph and the table was put to far away. We deleted a set paragraph to meet the requirements.

**Comments of the Reviewers:
Reviewer 1
Comment 1 Reviewer 1**: *When you do a sex-stratified analysis, you have to study the differences. If these differences are avoidable, then we would talk about "inequalities". Then we can talk about gender instead of sex. I think this terminology (e.g., in the title) should be better revised throughout the text.*
**Answer 1 R 1**: Thank you for your review of our manuscript and referring to a more precise wording. We changed the word “differences” to “inequalities” in the title and the manuscript as gender differences may be avoidable under other working conditions. We only kept the word “differences” when directly referring to numerical mean differences in the result section.

**Comment 2 Reviewer 1:** *As a small comment, in Table 3 (which puts Table 1) the Chi-squared column should be removed. This is a suggestion, as I do not think it adds anything. If the authors decide to leave this column, I have nothing to say.*
**Answer 2 R 1**: Thank you for pointing out how to make the manuscript more compact. We deleted the column as we agree that it does not add additional information.

**Reviewer 2
Comment 1 Reviewer 2:** *My main comment is that this study needs much more engagement with previous literature to motivate the examination of these gender differences by occupational subgroup. The paper currently focuses on how they differ in terms of gender composition, but there are other important differences between these subgroups. White collar and blue collar jobs have differences in physical job demands while low-skill and high-skill jobs presumably have large differences in terms of their psychosocial demands. These job characteristics could differentially impact men and women and should be discussed in both the intro to motivate the analyses and in the discussion to interpret the findings.*
**Answer 1 R 2**: Thank you for your review of our manuscript and pointing out the chance to include a wider range of perspective on the stresses that white- and blue-collar jobs. We additionally extended the following paragraph with additional literature as Doef (1999) Artazcoz (2016) and Ganster (2018) in the introduction (changes are highlighted in green):

“Regarding stresses, in blue-collar jobs, workers are more exposed to physical strains as chemicals and heavy weights [3, 17], while white-collar jobs rather lead to psychosocial and emotional demands which significantly lead to exhaustion and reduced well-being [3]. In addition to work intensity, emotional demands are the strongest predictor for impaired occupational health and burnout [18]. It is yet to be researched if these stresses differ in impairment on different genders. Overall, for all genders, conditions as longer and asocial working hours (especially more than fulltime), emotional demands, demands for hiding emotions, high demands and low control, discrimination, low job promotion, low predictability, low meaning of work low social support have been found to have a strong negative effect on psychosomatic complaints when comparing workers with more or less of these stresses with each other [3, 17, 19, 20, 21, 22, 23].”

Additionally, we did so in the discussion:
“Also, stresses differ between blue- and white-collar jobs as more physical stresses in blue-collar jobs [3, 17] and psychosocial and emotional stresses in white-collar [3]. It remains unclear if and why these inequalities in stresses and their connection of inequalities in psychosomatic complaints appear more in white-collar high-skilled jobs than in other occupational subgroups. There might be certain stresses, tasks and resources that predominantly influence psychosomatic complaints that women are more exposed to or react to more.”

**Comment 2 Reviewer 2:** *Can the authors provide citation(s) for how other studies have operationalized psychosomatic complaints? And some more background about* *why these complaints are likely to be affected by work?*
**Answer 2 R 2:** Thank you for pointing out to be more precise in the citation of our literature. To get a fuller impression of recent studies, we added the following paragraph in the introduction (changes highlighted in green):
“Previous research shows that women report more psychosomatic complaints at work than men as physical and emotional fatigue, sadness, irritability and muscle, heart, and stomach pain [1-5]. Papers used different styles of operationalisation as yes/no questions and sum scores of questionnaires, but the general trend was found again and again.”
Additionally, the paragraph for the introduction we presented in Comment 1 includes more information and literature where different working conditions were analysed in connection with complaints indicating that certain work(-ing conditions) leads to more or less complaints.

**Comment 3 Reviewer 2:** How was skill dichotomized into high vs low skill? **Answer 2 R 2:** The coding system of ISCO88 made job descriptions for a range of jobs and rated their skill requirements according to the work task that acquired the highest skill to do it (an additional information that was added in the manuscript as well). Occupations were grouped into several supergroups regarding skill and specialisation. To give a better insight which occupations were put where we adjusted the following paragraph in the methods section (changes highlighted in green):

“The sample divided into the occupational groups “white-collar high-skilled” (including legislators, senior officials, managers, professionals, technicians and associate professionals), “white-collar low-skilled” (including clerks, service workers, shop and market sales workers), “blue-collar high-skilled” (including skilled agricultural and fishery workers and craft and related trades workers) and “blue-collar low-skilled” (including plant and machine operators, assemblers and elementary occupations).”

Thus, skill wasn’t dichotomised per se but rated together with skill specialisation which resulted in the four occupational groups.

**Comment 4 Reviewer 2**: *Why were working hours categorized as “up to 10 hours worked per week”, “11-20 hours worked per week”, “21-30 hours worked per week”, and “30 or more hours worked per week”? Very few participants work 10 or fewer hours per week so it is unclear why this needs its own category. Would it be possible to divide the over 30 hours per week into multiple categories to identify those working long hours? Previous literature examining the effects of working hours on adverse outcomes has found more than full-time work to be a risk factor, yet the current categorization does not allow the authors to examine this.*

**Answer 4 R 2**: Thank you for pointing out the impact of working hours on stress. We added literature to these findings (e.g, Eurofound, 2017) and changed the subgroups into 5-hour-steps between 20 hours and 45+ hours to make the possible extra burden of working over 30 or 40 hours/ week better measurable. In the results section and discussion, we included the results of the regression analysis that our data validated the effect that longer working hours led to more complaints although the effect of collar remains when controlling for it.

**Comment 5 Reviewer 2:** *It would be clearer to refer to the “Child status” variable instead as “parental status”.* **Answer 5 R2:** To clarify the wording, we changed the words in the text as suggested.

**Comment 6 Reviewer 2**: *The statistical analyses need some sort of correction for multiple testing. The analyses presented in Table 3 represent 28 comparisons (7 symptoms x 4 occupational subgroups), so it is not surprising that several are statistically significant.*
**Answer 6 R2**: To improve the quality of the analysis, we added a Bonferroni correction using a stricter α-level in the analysis. Using the formular α_corrected_= α_chosen a priori_ / n with n= 7x4= 28 comparisons. This resulted in α_corrected_= 0.05 / 28 = 0.002. 0.002 was used as a stricter cut-off for significance. Some values did not meet the significance anymore, the overall trend and statement that white-collar high-skilled female workers are dominantly under complaint inequalities remained.

**Comment 7 Reviewer 2**: *Throughout the results section, the interpretations of the odds ratios are incorrect and overstate the findings. For example, the interpretation of an odds ratio of 2.08 as “women were 2.08 times more likely to report headaches as compared to men” should be “women had 2.08 times the odds of reporting headaches as compared to men.”*
**Answer 7 R2**: Thank you for pointing out this error of interpretation. We adjusted the wording in the result section as suggested.
